# Cytological changes in oral mucosa induced by smokeless tobacco

**DOI:** 10.18332/tid/109544

**Published:** 2019-05-28

**Authors:** Mohammed E. A. Mohammed, Eid I. Brima

**Affiliations:** 1 Department of Chemistry, Faculty of Science, King Khalid University, Abha, Saudi Arabia; 2 School of Allied Health Sciences, Faculty of Health and Life Sciences, De Montfort University, Leicester, United Kingdom

**Keywords:** local Saudi snuff, cytological changes, keratinization, binucleation, dual abnormalities

## Abstract

**INTRODUCTION:**

This study investigated the cellular changes in the mouth of smokeless tobacco (shamma) users among Saudi citizens from the Najran region.

**METHODS:**

Healthy 61 shamma users and healthy 61 non-users participated in this study after informed consent. A mouth swap was taken from each study subject and investigated for cellular changes using a Papanicolaou stain. The results were analyzed using the t-test per cent of the StatPac statistical program.

**RESULTS:**

Keratinization, binucleation and combined results were associated with shamma use. The number of shamma users with abnormal histology was 14 (22.9%) (p≤0.0001). The shamma types associated with the abnormal cytology were: Suhaily (9/36; p=0.0014), black (2/9; p=0.08), white (2/6; p=0.41), and red (1/1; p=1.00). The percentage of the tissues with abnormal histology decreased with the increase in the duration and frequency of smokeless tobacco use, except for the white shamma which increased with the increase in the frequency.

**CONCLUSIONS:**

Shamma use affected the mouth epithelial cytology of 22.9% of its users. The histological changes were inflammation, keratinization, binucleation, and atypia. The Suhaily smokeless tobacco was associated with the highest percentage of the abnormal cytology results, while the duration and frequency of smokeless tobacco use decreased the number of tissues with abnormal histology.

## INTRODUCTION

Smokeless tobacco is well known to be associated with mouth diseases, including leukoplakia, erythroplakia, and oral submucous fibrosis^1^. Leukoplakia, erythroplakia and oral submucous fibrosis are associated with cellular changes such as keratinization, enlarged nucleus, atypia, multi-nucleation, and inflammatory reaction^2-6^. All of the above smokeless tobacco-induced changes are considered as characteristics of carcinogenesis and metastasis^7,8^.

Smokeless tobacco in Saudi Arabia is known as shamma, prepared as a mixture of powdered unburned leaves from *Nicotiana tabacum* alone or mixed with leaves from *Nicotiana rustica, Nicotiana glauca,* carbonate of lime, ash, black pepper, oils, and flavorings. There are many types of shamma according to color (black, white, red, grey, brown, green, yellow) and origin: Suhaily, Afzal (Indian origin), Maswar (Afghanistan), and Toombak (Sudan)^9^.

Smokeless tobacco, including shamma, contains at least 30 chemicals that are associated with high cancer risk, including Tobacco-Specific Nitrosamines (TSNA), while the correlation between heart disease and blood pressure with smokeless tobacco is well known^10,11^.

Changes in oral cellular appearance under the microscope are mostly due to malignancy, infection, infarction or necrosis, chemotherapy, and radiotherapy^12^. Atypical cellular changes can be seen in the cases of infarction, necrosis, and haemorrhage^13^. Hence, the aim of this study was to investigate the effect of shamma on the appearance of oral mucosa cells.

## METHODS

Sixty-one shamma users and 61 non-users among Saudi citizens from the Najran region were involved in this study. The cellular effects were studied of eight shamma types: Suhaily, Afzal, white, black, brown, grey, red, and yellow. All the study subjects were males of age 18–80 years for shamma users and 19–88 years for non-users. The age range was divided into three groups: 18–44, 45–64 and ≥65 years. The duration of shamma use was divided to two groups: 1–5 and 6–10 years; and the frequency (times/day) of use was categorized into three groups: 1–5, 6–10, and 11–15. This study was conducted after approval from the ethical committee of King Khalid University. The study subjects were involved after signing an informed consent.

Cytological smears from oral mucosa were collected from the study participants. The study subjects were told to wash their mouth with normal saline solution before the sampling process. The dipping site was dried with a smooth wipe to expel excess saliva. A smooth brush was used to scratch the site of snuffing and the tongue two times to ensure the collection of the samples from the inner layers of the oral mucosa. Each collected sample was smeared on a slide and fixed in 96% ethanol for 30 minutes. The fixed buccal smears were stained with the Papanicolaou stain^14^. The Papanicolaou stained slides were investigated using the 40× magnification of an Olympus light microscope. The cells were considered as atypia if they had: an irregular shape; hyperchromatism; irregular nuclear shape, size or border; and irregular cytoplasm size. Presence of two nuclei in the buccal cells was considered as binucleation, while accumulation of white blood cells was the marker of inflammation, and keratinization was confirmed by the yellow staining of keratins, the intermediate filament proteins.

Statistical analysis was done using the StatPac program for basic and advanced statistics. Comparison between the control and the cases was performed by the t-test per cent. The significance level was set at 0.05.

## RESULTS

The cellular changes induced by eight shamma types were studied. The eight shamma types and their use frequencies in the study population were: Suhaily (36), black (9), white (6), grey (4), yellow (3), red (1), afzal (1), and brown (1).

**Figure 1:**
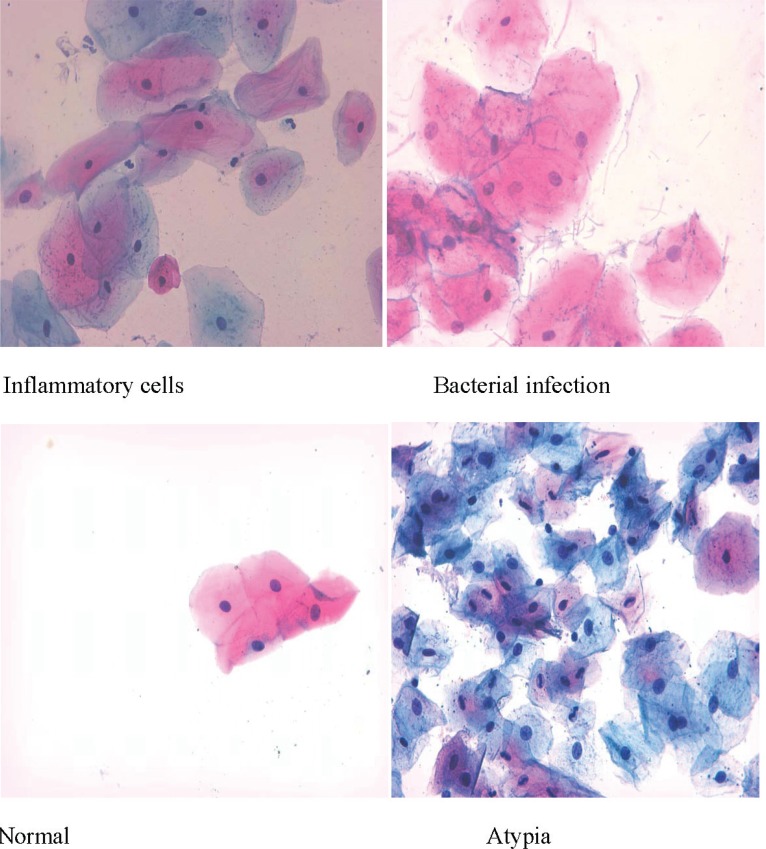
Representative non-shamma associated Papanicolaou staining results

**Figure 2:**
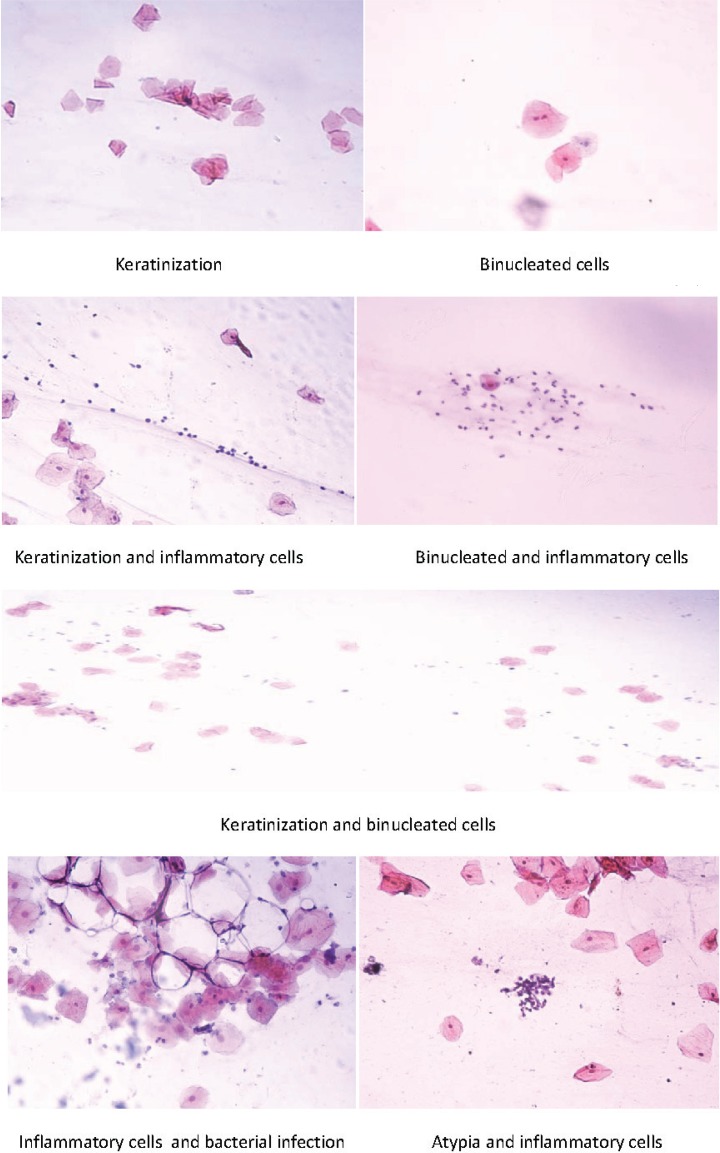
The oral mucosa cytological changes associated with Saudi smokeless tobacco

The interpretation of the Papanicolaou staining results showed the presence of eleven results with different percentages: normal, inflammatory cells, bacterial infection, atypia, keratinization, binucleation; and five combinations: atypia and inflammatory cells, keratinization and inflammatory cells, bacterial infection and inflammatory cells, binucleation and keratinization, and binucleation and inflammatory cells (Table 1 and Figures 1 and 2). Four results were seen in the oral histological samples of the shamma users and non-users: normal cells, inflammatory cells, atypia, and bacterial infection (Figure 1). The absolute numbers and percentages of each result are presented in Table 1. However, the percentages of the tissues with normal histology in the shamma users and non-users were 50.8% and 68.9%, respectively (p=0.043) (Table 1). The percentages of the abnormal results in the shamma users and non-users were 49.2% and 31.1%, respectively (p<0.001). The other seven results were associated with the use of shamma (not seen in the histology samples of the non-users) (Figure 2). Four shamma types induced the seven results: Suhaily, black, white and red (Table 2). Of the shamma associated abnormal cytology results, 64.3% were due to the Suhaily type (9/14; p=0.28), while 25% (9/36; p=0.0014) of the Suhaily shamma were associated with the cellular changes (Table 2).

**Table 1:** The Papanicolaou staining results of the shamma user and non-user study subjects (N=122)

	*Papanicolaou staining result*	*Shamma user*	*Shamma non user*	*P (t-test percent)*
		*n (%)*	*n (%)*	
1	Normal^a^	31 (50.8)	42 (68.9)	0.043
2	Inflammatory cell^a^	14 (23)	15 (24.6)	0.84
3	Bacterial infection^a^	1 (1.6)	3 (4.9)	0.31
4	Atypia^a^	1 (1.6)	1 (1.6)	1.00
5	Keratinization^b^	3 (4.9)	0	-
6	Binucleated^b^	2 (3.3)	0	-
7	Atypia + inflammatory cells^b^	2 (3.3)	0	-
8	Keratinization ^+^ inflammatory cells^b^	3 (4.9)	0	-
9	Inflammatory cells ^+^ bacterial infection^b^	2 (3.3)	0	-
10	Binucleation ^+^ keratinization^b^	1 (1.6)	0	-
11	Binucleation ^+^ inflammation^b^	1 (1.6)	0	-
Total		61 (100)	61 (100)	-

a Staining results seen in the shamma users and non-users.

b Staining results associated with shamma use.

**Table 2:** Shamma types and their Papanicolaou staining results (N=61 )

*SN*	*Shamma type*	*n*	*Shamma associated abnormal results*		*p*
			*n*	*%*	
1	Suhaily	36	9	25	0.0014
2	Black	9	2	22.2	0.08
3	White	6	2	33.3	0.43
4	Grey	4	0	0	--
5	Yellow	3	0	0	--
6	Red	1	1	50	1.00
7	Brown	1	0	0	-
8	Afzal	1	0	0	-
Total		61	14	22.95	≤0.0000

The association between the duration and type of shamma use is presented in Table 3. White shamma use induced the incidence of the abnormal cytology by the increase in the use frequency, unlike the results of Suhaily and black shamma.

**Table 3:** Effect of duration and frequency of shamma use on mouth cytology

	*Type of shamma*		*Normal*	*Abnormal*	*Total*
**Duration** (years)	Suhaily	1–5	14	6	20
		6–10	13	3	16
	Black	1–5	4	2	6
		6–10	3	0	3
	White	1–5	2	1	3
		6–10	2	1	3
**Frequency** (times/day)	Suhaily	1–5	16	7	23
		6–10	8	2	10
		11–15	3	0	3
	Black	1–5	6	2	8
		6–10	1	0	1
		11–15	0	0	0
	White	1–5	2	0	2
		6–10	0	1	1
		11–15	2	1	3

**Figure 3:**
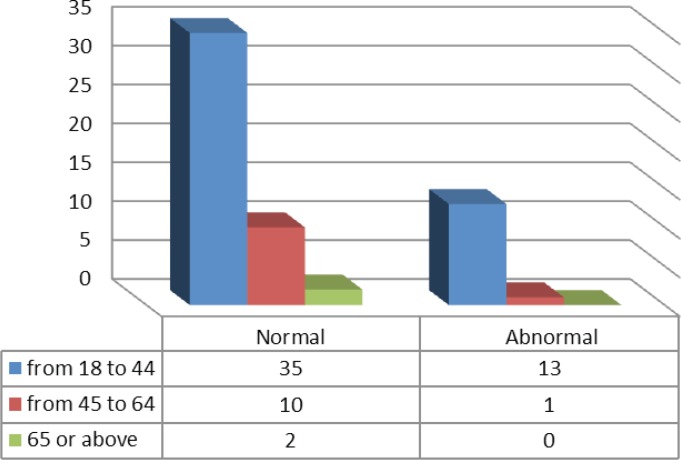
The Pap stain cytology results of the participants within the different age groups

The majority of the shamma associated cytology (13/14) were seen in the tissues from the participants of the age group 18–44 years, while only one abnormal result belonged to the participants of the age group 45–64 years (Figure 3).

## DISCUSSION

In this study smokeless tobacco (shamma) use was found to be associated with three abnormal cytology results: keratinization, binucleation, and combinations such as atypia and inflammation. The abnormal mouth cytology results were driven by the type of smokeless tobacco rather than the duration or frequency of smokeless tobacco use or the age of the dippers.

Previous research found that keratinization was found in all the scrapes of oral mucosa from Sudanese Toombak (Sudanese smokeless tobacco) dippers. Ahmed et al.^15^ found that 53% of their study population was characterized by excessive keratinization, a fact also found by other researchers^11,15,16^. Surprisingly, smokeless tobacco-induced keratinization has a benefit: it prevents aphthous stomatitis of the oral mucosa. Aphthous stomatitis is a painful recurrent ulcer of unknown cause^17^. Keratinization is one of the early indicators and poor prognosis of oral squamous cell carcinoma^18,19^, and may be involved in the aetiology of oral cancers.

In accordance with our findings, Indian studies showed the presence of binucleated buccal mucosa cells in smokeless tobacco users^20,21^. Chen^22^ in 1989 treated 30 rats with smokeless tobacco for one year and observed that the smokeless tobacco-induced formation of numerous binucleate spinous cells. Many studies correlated dysplasia and malignancy lesions in the oral mucosa with the use of smokeless tobacco^11,16,23-25^. An *in vitro* study mentioned that smokeless tobacco might induce inflammatory reactions through recruitment of leucocytes in the smokeless tobacco use sites^25^. Studies from Uzbekistan and India found that smokeless tobacco use is associated with inflammation, alveolar bone damage, dysplasia, and squamous cell carcinoma. Smokeless tobacco was also correlated with an increased blood level of IL-12^26,27^. However, oral inflammation is an important consequence of oral squamous cell carcinoma progression^28^.

Regarding the effect of the duration and frequency of smokeless tobacco use, many studies found that the duration and frequency of smokeless tobacco use were associated with cytological changes and malignant transformations^1,5,29^. Similar to our findings, Daniels et al.^30^ found that the duration and frequency of smokeless tobacco use are not associated with epithelial changes while they are associated with smokeless tobacco type.

The similarities and differences between the findings of this study and previous studies may be due to the ethnic origin and the different types of smokeless tobacco. Differences were reported when the findings of this study were compared to African studies, whereas similarities were seen when the findings of this study were compared to studies from Asia and the Indian peninsula.

### Limitations

Our study has a number of limitations, including the small number of samples overall and within subgroups assessed. Moreover, by design this study cannot determine the causes of the abnormal results, while this study did not quantify the abnormalities such as the number of binucleated cells so as to find an association between them and the type of smokeless tobacco, duration and frequency of use, and age of users. To overcome the limitations of this study, more comprehensive research with a large number of samples are planned for the near future.

## CONCLUSIONS

Oral mucosa histological changes associated with smokeless tobacco (shamma) use in Saudi Arabia were found to be inflammation, keratinization, binucleation, and atypia.

## CONFLICTS OF INTEREST

The authors have completed and submitted the ICMJE Form for Disclosure of Potential Conflicts of Interest and none was reported.
